# Frequency, outcomes, and need for intervention in stricturing gastrointestinal tuberculosis: a systematic review and meta-analysis

**DOI:** 10.1186/s12876-023-02682-x

**Published:** 2023-02-23

**Authors:** Anuraag Jena, Ritin Mohindra, Kirtan Rana, Pardhu B. Neelam, Dhuni Chand Thakur, Harjeet Singh, Pankaj Gupta, Vikas Suri, Vishal Sharma

**Affiliations:** 1 Department of Gastroenterology, Postgraduate Institute of Medical Education and Research, Chandigarh, 160012 India; 2 Department of Internal Medicine, Postgraduate Institute of Medical Education and Research, Chandigarh, 160012 India; 3 Department of Community Medicine and School of Public Health, Postgraduate Institute of Medical Education and Research, Chandigarh, 160012 India; 4 Department of Surgical Gastroenterology, Postgraduate Institute of Medical Education and Research, Chandigarh, 160012 India; 5 Department of Radiodiagnosis, Postgraduate Institute of Medical Education and Research, Chandigarh, 160012 India

**Keywords:** Intestinal tuberculosis, Crohn's disease, Tuberculous peritonitis, Peritoneal tuberculosis, Gastrointestinal tuberculosis, Abdominal tuberculosis

## Abstract

**Background:**

Gastrointestinal strictures impact clinical presentation in abdominal tuberculosis and are associated with significant morbidity.

**Aim:**

To conduct a systematic review of the prevalence of stricturing disease in abdominal and gastrointestinal tuberculosis and response to antitubercular therapy (ATT).

**Methods:**

We searched Pubmed and Embase on 13th January 2022, for papers reporting on the frequency and outcomes of stricturing gastrointestinal tuberculosis. The data were extracted, and pooled prevalence of stricturing disease was estimated in abdominal tuberculosis and gastrointestinal (intestinal) tuberculosis. The pooled clinical response and stricture resolution (endoscopic or radiologic) rates were also estimated. Publication bias was assessed using the Funnel plot and Egger test. The risk of bias assessment was done using a modified Newcastle Ottawa Scale.

**Results:**

Thirty-three studies reporting about 1969 patients were included. The pooled prevalence of intestinal strictures in abdominal tuberculosis and gastrointestinal TB was 0.12 (95%CI 0.07–0.20, I^2^ = 89%) and 0.27 (95% CI 0.21–0.33, I^2^ = 85%), respectively. The pooled clinical response of stricturing gastrointestinal tuberculosis to antitubercular therapy was 0.77 (95%CI 0.65–0.86, I^2^ = 74%). The pooled stricture response rate (endoscopic or radiological) was 0.66 (95%CI 0.40–0.85, I^2^ = 91%). The pooled rate of need for surgical intervention was 0.21 (95%CI 0.13–0.32, I^2^ = 70%), while endoscopic dilatation was 0.14 (95%CI 0.09–0.21, I^2^ = 0%).

**Conclusion:**

Stricturing gastrointestinal tuberculosis occurs in around a quarter of patients with gastrointestinal tuberculosis, and around two-thirds of patients have a clinical response with antitubercular therapy. A subset of patients may need endoscopic or surgical intervention. The estimates for the pooled prevalence of stricturing disease and response to ATT had significant heterogeneity.

**Supplementary Information:**

The online version contains supplementary material available at 10.1186/s12876-023-02682-x.

## Introduction

Abdominal tuberculosis is an important form of extra-pulmonary tuberculosis. It has a varied clinical presentation depending on the site of involvement: peritoneum, intestines, visceral organs, and/or abdominal lymph nodes. Tuberculous peritonitis and gastrointestinal tuberculosis (GITB) are the two most frequent patterns. The ileocecal region is the most common site of tuberculosis involvement in the intestine (25 to 90%). The morphologic patterns of GITB include ulcerative, hypertrophic, stricturing, or a combination of these. [1, 2] For the purpose of this systematic review we have used the ‘abdominal tuberculosis’ as an umbrella term that encompasses both the luminal (intestinal or gastrointestinal tuberculosis) and peritoneal tuberculosis (tuberculous peritonitis). While strictures are more frequent in intestinal tuberculosis, they may also occasionally occur in peritoneal tuberculosis due to peritoneal fibrosis and adhesions.

*Mycobacterium tuberculosis*, upon penetration of the intestinal mucosa, initiates a local inflammatory reaction in the submucosal lymphoid tissue. This leads to lymphangitis, granuloma formation, caseation necrosis, mucosal ulceration, and scarring [3]. The clinical presentation of abdominal tuberculosis depends on the underlying morphology: extensive ulcerations are usually associated with diarrhea, while stricture and hypertrophic forms may present with abdominal pain and intestinal obstruction features [1–4]. The reasons for the predominance of a particular morphologic pattern in an individual patient are unclear. Recurrent episodes of pain and obstruction may lead to frequent hospitalizations, poor quality of life, and the need for surgical interventions amongst this subset of patients. Gastrointestinal strictures are reported in a variable number of patients with tuberculosis: the variations are due to differing populations (intestinal or peritoneal or both) or selection bias (surgical series versus medically managed patients) in the published reports. Strictures in GITB may be inflammatory or fibrotic, depending predominantly on the activity and duration of the disease. Response of the intestinal strictures to anti-tubercular therapy (ATT) is varied as the inflammatory component may get resolved with treatment but also lead to healing and scarring with subsequent persistence of the fibrotic stricture. The response of tubercular strictures to ATT could be a clinical response (resolution of symptoms of stricture like intestinal obstruction or pain) or stricture response (resolution of stricture as assessed using radiology or endoscopy).

Therefore, we planned a systematic review to study the frequency of stricturing GITB in patients with abdominal TB and GITB, response to ATT and need for intervention (endoscopic dilatation or surgery) in these patients.

## Methods

This meta-analysis was conducted in accordance with the Meta-analysis Of Observational Studies in Epidemiology (MOOSE) group recommendations and Preferred Reporting Items for Systematic Reviews and Meta-Analyses (PRISMA) guidance. [5, 6]

### Search strategy

We searched Pubmed and Embase for articles reporting on frequency, clinical outcomes, and the need for intervention (surgery or endoscopic dilatation) in patients with stricturing gastrointestinal tuberculosis. The search was recent till 13th January, 2022. The search strategy combined the terms “Intestinal Tuberculosis” OR “Gastrointestinal Tuberculosis” OR “Peritoneal Tuberculosis” OR “Tuberculous peritonitis” OR “Abdominal Tuberculosis” with 'stricture’ OR ‘fibrosis’ OR ‘stenosis’ OR ‘surgery' using the operator ‘AND’. The detailed search strategy is depicted in Additional file 1: Table S1. The results were combined, and duplicates were removed. The title and abstract screening were done by two reviewers (RM and KR) independently. The titles selected underwent full-text screening.

### Study selection and data extraction

All articles, irrespective of article type or the language of publication, which provided data relevant to the study question were included. This included one or more of the following.

1. Frequency of intestinal strictures or stricturing disease in patients with intestinal or abdominal tuberculosis
2. Frequency of clinical response, stricture improvement (as determined using radiological and endoscopic assessment) in stricturing intestinal tuberculosis

We excluded studies that reported on a series of < 10 patients, those which did not provide clear data for stricturing disease separately, and series which reported predominantly or solely on surgically managed patients and studies. For each planned analysis, we excluded those studies with a total patient number of 5 or less eligible for that analysis. We also excluded those study types which did not provide original data like reviews, letters, and guidelines. Abstracts were included if they provided relevant information.

The data were extracted from each of the studies for the type of study population (abdominal TB or intestinal TB or both), mean age and gender, frequency of stricturing disease in the subset of abdominal TB and gastrointestinal tuberculosis, clinical response (and its definition) to antitubercular therapy (ATT), stricture resolution (endoscopic or radiologic) and requirement of intervention (surgery or endoscopic balloon dilatation). Data extraction was done by two reviewers independently (AJ, RM) and any discrepancies were resolved by mutual discussion with a third reviewer (VS).

### Definitions

For the purpose of this systematic review, we have used the ‘abdominal tuberculosis’ as an umbrella term that encompasses both the luminal (intestinal or gastrointestinal tuberculosis) and peritoneal tuberculosis (tuberculous peritonitis). Gastrointestinal tuberculosis specifically refers to intestinal (i.e. luminal) involvement.

### Outcomes

We calculated the pooled prevalence of stricturing GITB in patients with abdominal TB. We also calculated the pooled prevalence of stricturing GITB in patients with intestinal TB. We calculated pooled clinical response rate and pooled stricture response (endoscopic and radiologic) rates after ATT. We calculated the pooled rates of intervention required in stricturing GITB i.e. surgery or endoscopic dilatation.

### Analysis

We used the R statistical software version 4.1.2 for the analysis and in addition to the base package, meta and metafor packages were used. [7, 8] We calculated the pooled prevalence rates using a random effect method with an inverse variance approach. Logit transformations were made for the individual rates before computation of the pooled summary.

The heterogeneity was assessed using the I^2^ statistic, and heterogeneity of > 50% was considered as high. We performed subgroup analyses based on the site of disease, type of studies (prospective, retrospective), and the duration of ATT to evaluate the heterogeneity. Sensitivity analysis was also performed after excluding studies with a high or fair risk of bias. Baujat plots were constructed to identify studies contributing to heterogeneity.

### Risk of bias

Two of the investigators (AJ and PB) independently assessed the methodological quality and risk of bias of studies using a modified Newcastle Ottawa Scale. [9] Any discordance in risk of bias, was settled with mutual agreement with a third reviewer (VS). Since no comparative analyses was performed for this proportional meta-analysis, we removed the comparability domain in the modified scale. We considered only those studies to be of good quality if the score was seven. Publication bias was assessed using Funnel plot (standard and Trimfill) and Egger test. [10]

## Results

### Study selection

**Fig. 1:**
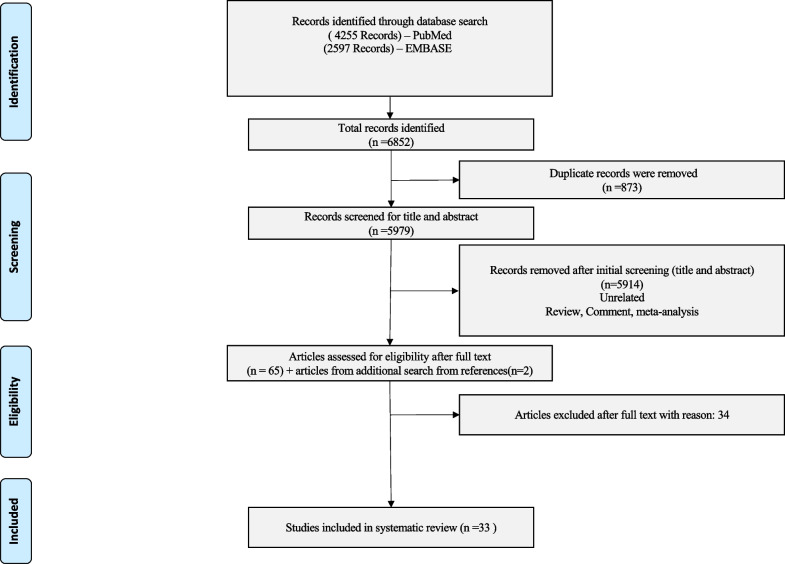
The PRISMA flow chart showing the process of screening and selection of eligible studies

The result of the search yielded 6852 citations. (Fig. 1, PRISMA flow chart) Of the total of 6852 studies, there were 873 duplicates. We excluded 5914 citations after the abstract screening, and 65 citations were screened for full text. We obtained 2 further studies after manually searching the references of included studies. After full-text screening, we excluded 34 studies that did not fulfill the inclusion criteria. Eventually, 33 studies (30 full texts and 3 abstracts) were included in the final analysis. The details of the included studies are illustrated in Table 1.[11–43] The details of the excluded studies are illustrated in Additional file 1: Table S2.

**Table 1:** Details of the included studies in the meta-analysis

Authors with year	Country	Type of study	ATB and GITB	Associated Extra–intestinal involvement	Duration of symptoms	Associated conditions	Stricturing TB (n)	Age, Number of males	Location	Clinical features	Clinical Response	ATT	Endoscopic Intervention	Surgery	Stricture resolution	Stricture Response
Anand BS et al., 1998	India	Prospective	–	–	24.4 (33.3) months	–	39	33.4 ± 15.1yrs, 25	Duodenum (n = 1), Small intestine (n = 15), Ileocecal area (n = 10), Colon (n = 9), multiple sites (n = 4)	Pain (n = 36), Obstruction(n = 34), weight loss (n = 36), diarrhea (n = 12), bleeding(n = 4)	31 of 34	3 months HRS + 9 months HR (E if reaction to any)	–	3	16 of 23	
Alvares JF et al., 2005	India	Retrospective	GITB (n = 43)	11	–	–	10	–	Colonic (n = 10)	–	8 of 10	2 months HRZES + 7 months months HR	–	2	8 of 10	
Aggarwal P et al., 2017	India	Ambispective	GITB (n = 286)	–	12 (6–24) months	–	128	Median age: 35 years(n = 106), 63	Duodenum (n = 4), Small intestine (n = 10), Ileocecal area (n = 52), Colon (n = 37), Multiple sites (n = 4)	Fever (n = 44), Pain (n = 99), Obstruction (n = 80), Weight loss(n = 86)	52 of 104	2 months HRZE + 4–7 months HR	12	7	25 of 106	–
Amrapurkar DN et al., 2008	India	Prospective	GITB (n = 26)	6	7.2 (3.4) months	–	5	–	–	–	1 of 5	2 months HRZE + 10 months HR	0	4	1 of 5	–
Bhargava DK et al. 1992	India	Retrospective	GITB (n = 29)	10	–	–	10	Mean age– 38.4 years, Males (n = 4)	Colonic (n = 10)	–	7 of 10	Regimen not mentioned	–	3	–	–
Cheng W et al. 2019	China	Retrospective	GITB (n = 49)	Hepatic– 8, Cervical TB–4, Renal–3, Ovarian–1	102 (3–7300) days	3 on immunosuppressants	11	–	–	–	–	–	–	–	–	–
Das HS et al., 2000	India	Retrospective (abstract)	GITB (n = 21)	–	–	–	3	–	Colonic (n = 3)	–	–	–	–	–	–	–
Deka UJ et al., 2012	India	–(abstract)	GITB (n = 44)	–	–	–	7	–	–	–	7 of 7	6 months course	–	–	–	–
Dutta AK et al., 2011	India	Prospective	GITB (n = 24)	–	3 months (1 month to 2 years)	–	4	–	–	–	–	–	–	1	–	–
Fillion A et al., 2015	France	Retrospective	ATB (n = 21), GITB (n = 7)	–	13 months	2 coexisting immunosuppressed conditions	1	–	–	–	–	6 months four drug regimen	–	1	–	–
Gan H et al., 2016	China	Retrospective	GITB (n = 81)	TB pleuritis: 14, lymph node TB: 7, uro– genital TB: 4, bone TB: 2	8 months	–	16	–	–	–	–	3 months HRZE + 9–15 months HR	–	–	–	–
Hu ML et al., 2009	Taiwan	Retrospective	ATB(n = 14), GITB (n = 3)	TB meningitis– 3	–	1 had Cancer	1	–	–	–	–	–	–	–	–	–
Jung Y et al., 2016	South Korea	Retrospective	GITB (n = 98)	–	–	2 had malignancy	9	–	–	–	–	–	–	–	–	–
Kentley J et al., 2017	UK	Retrospective	ATB (n = 147), GITB (n = 61)	Appendiceal TB–2	13 (2–16) weeks	–	9	–	–	–	–	–	–	–	–	–
Khan R et al., 2006	Pakistan	Retrospective	ATB (n = 209), GITB (n = 102)	Past TB– 13	–	0	17	–	–	–	–	9–12 months various combination of HRZES	–	11	–	–
Kim KM et al., 1998	Korea	Retrospective	GITB (n = 42)	–	–	–	3	–	–	–	–	–	–	–	–	–
Larsson G et al., 2015	India	Prospective	GITB (n = 30)	–	–	–	3	–	–	–	–	–	–	–	–	–
Lee YJ et al., 2006	Korea	Prospective	GITB (n = 44)	–	–	–	8	–	–	–	–	–	–	–	–	–
Lu S et al., 2020	China	Retrospective	GITB (n = 10)	–	–	–	6	–	–	Pain (n = 6)	–	3 months intensive + 9–15 months consolidation	–	2	–	–
Lu Y et al., 2021	China	Retrospective	GITB (n = 84)	–	–	–	24	–	–	–	–	–	–	–	–	–
Makanjuola D et al., 1998	Saudi Arabia	Retrospective	GITB (n = 21)	liver and pancre in one and pancreas alone in one patient	–	–	8	–	–	Obstruction (n = 6), Diarrhea (n = 1)	–	–	–	–	–	–
Millar AJW et al., 1990	South Africa	Retrospective	ATB(n = 95) children	–	–	Factor V deficiency in one	16	–	–	–	–	Quadruple HRZE till discharge or TB hospital + triple therapy total 6 months	–	4	–	–
Misra SP et al., 1999	India	Retrospective	GITB (n = 50)	Already on ATT: 13 patients	–	0	12	–	Colonic (n = 12)	–	10 of 12	2 months HRZE + 10 months HR	1	2	4	10
Mukewar S et al., 2012	India	Prospective	GITB (n = 69)	–	–	0	30	–	Colonic (n = 30)	–	26 of 30	2 months HRZE + 7 months HR	0	4	16 of 21	17 of 21
Nagi B et al., 2003	India	Retrospective	GITB (n = 74)	10	–	0	40	–	Colonic (n = 40) Transverse–20 Rectum–13 Ascending–9 Sigmoid–3	–	–	–	–	–	–	–
Palmer KR et al., 1985	UK	Retrospective	ATB (n = 90), GITB (n = 42)	Liver– 8	4 ± 0.9 years	–	10	–	–	–	–	Variable regimens of HRZE and PAS	–	–	–	–
Singh H et al., 2018	India	Retrospective	ATB (n = 119), GITB (n = 75)	Prior ATT: 16	–	0	48	–	–	–	26 of 48	2 months HRZE + 4 months HRE	10	14	–	–
Singh V et al., 1996	India	Retrospective	GITB (n = 62)	–	15 days– 10 years	–	17	–	Colonic (n = 17)	–	17	Standard regimen	–	–	–	–
Sinha S et al., 2017	India	Prospective (abstract)	GITB (n = 32)	–	–	–	13	–	–	–	9 of 13	9 months ATT	–	4	–	–
Tripathi PB et al., 2009	India	Retrospective	GITB (n = 110)	14	–	–	57	–	–	–	–	–	–	–	–	–
Udgirkar S et al., 2019	India	Prospective	ATB (n = 176), GITB (n = 162)	TB Meningitis	165 + /–23 days	–	29	–	Terminal ileum (n = 6), Ileocecal area (n = 13), Ascending colon (n = 4), Hepatic flexure (n = 2) Transverse colon (n = 3), Descending (n = 1)	–	23 of 28	6 months four drug followed by two, MDR–total 18 months with second line	3	2	3	21
Uygur–Bayramicli O et al., 2003	Turkey	Prospective	ATB (n = 31), GITB (n = 19)	Bone TB–2	1 month–1 1 years	–	1	–	Colon (n = 1)	–	–	Four drug regimen for 9 months	–	–	–	–
Zhu QQ et al., 2014	China	Retrospective	GITB (n = 35)	–	–	–	22	–	–	–	–	–	–	–	–	–

*ATB* Abdominal Tuberculosis, *ATT* Anti-tubercular therapy, *E* Ethambutol, *GITB* Gastro-intestinal Tuberculosis, *H* Isoniazid, *MDR* Multidrug resistant, *PAS* Para-aminosalicylic acid, *R* Rifampicin, *S* Streptomycin, *Z* Pyrazinamide

### Prevalence of stricturing GITB

**Fig. 2:**
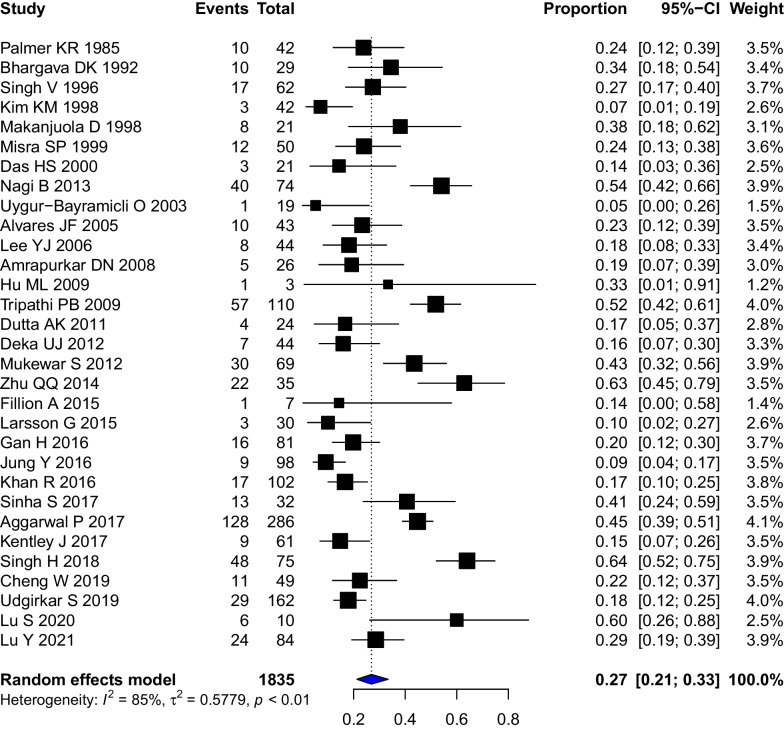
Forest Plot showing the pooled prevalence of stricturing disease in patients with gastro-intestinal tuberculosis

Overall, 9 studies (902 patients) reported the frequency of stricturing GITB in the setting of abdominal TB. The pooled prevalence of intestinal strictures in abdominal TB was 0.12 (95% CI 0.07–0.20, I^2^ = 89%) (Additional file 1: Fig. S1) (Fig. 2).

**Fig. 3:**
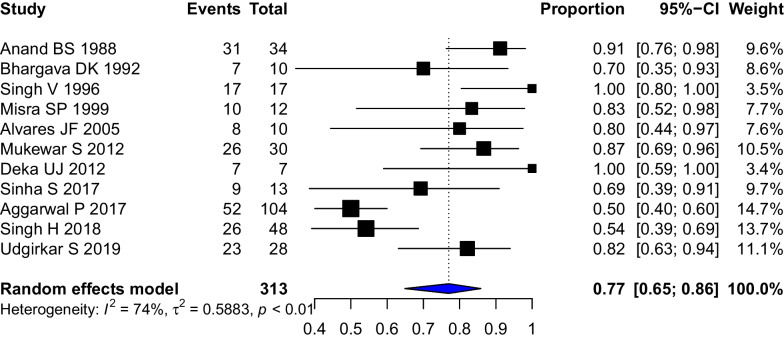
Forest Plot showing the pooled clinical response rates to anti-tubercular therapy in patients with stricturing gastrointestinal tuberculosis

For gastrointestinal tuberculosis, 31 studies (1835 patients) reported the frequency of intestinal strictures. The pooled prevalence of intestinal strictures in gastro-intestinal TB was 0.27 (95%CI 0.21–0.33, I^2^ = 85%) (Fig. 3). The Baujat plot constructed for studies suggested that the studies by Singh H et al. 2018, Jung Y et al. 2016, Agarwal P et al. 2017 contributed the maximum to the heterogeneity (Additional file 1: Fig. S2). [13, 23, 37] However, for the lack of clear reasoning to exclude these we did not perform a sensitivity analysis after removing these studies. To evaluate heterogeneity, we conducted a subgroup analysis by stratifying the studies by stricture site. However, the heterogeneity remained high. There were 7 studies (348 patients) reporting the frequency of strictures in colonic tuberculosis. The pooled prevalence of stricturing disease in colonic TB was 0.32 (95%CI 0.23–0.43, I^2^ = 74%) (Additional file 1: Fig. S3). Subgroup analysis based on the study types found that one study with an unclear design had the lowest prevalence (0.16, 0.7–0.30) while one with ambispective design had the highest prevalence (0.45, 0.39–0.51) of stricturing disease. The prevalence of strictures was higher in retrospective studies (0.29, 0.21–0.37) and compared to prospective studies (0.22, 0.14–0.32) (Additional file 1: Fig. S4). The subgroup analysis on the basis of the duration of ATT did not suggest any differences in stricturing disease (*P* = 0.9677) (Additional file 1: Fig. S5).

Sensitivity analysis by including only the six studies deemed low risk of bias, suggested that pooled estimates of stricturing disease were similar (0.36, 95% CI 0.24; 0.49) **(**Additional file 1: Fig. S6).

### Response to therapy

**Fig. 4:**
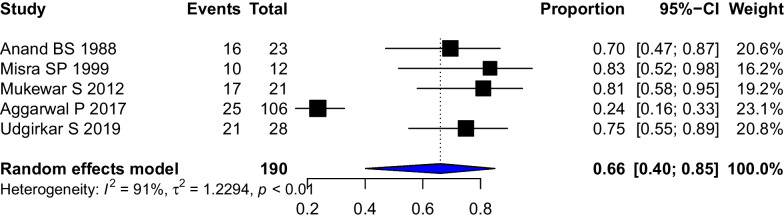
Forest Plot showing the pooled stricture response rates to anti-tubercular therapy in patients with stricturing gastrointestinal tuberculosis

**Fig. 5:**
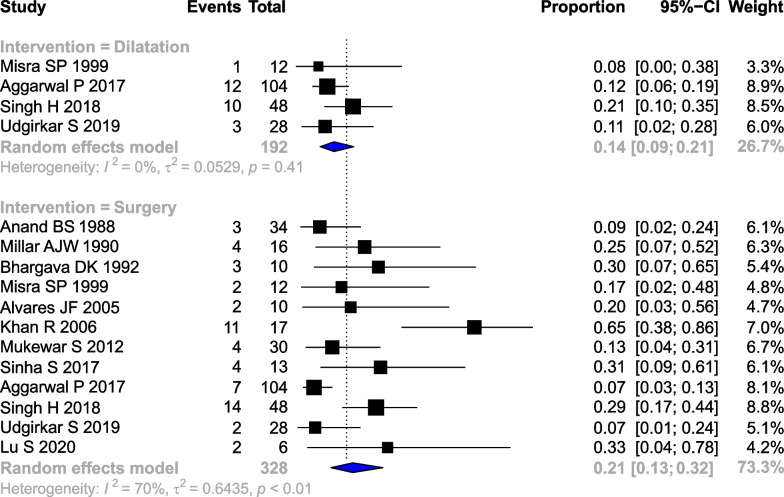
Forest plot showing the pooled rates of **a** endoscopic dilatation **b** surgery in patients with stricturing gastrointestinal tuberculosis

The definitions of clinical response and clinical cure in each study have been provided in Additional file 1: Table S3. For the purpose of analysis, we used the clinical response rates wherever available. Eleven studies (313 patients) of tubercular intestinal strictures reported clinical responses to therapy. The pooled clinical response of strictures to therapy was 0.77 (95%CI 0.65–0.86, I^2^ = 74%) (Fig. 4). The stricture response/resolution as defined on the basis of endoscopic or radiological criteria in each study has been provided in Additional file 1: Table S3. The pooled stricture response rate (5 studies, 190 patients) was 0.66 (95%CI 0.40–0.85, I^2^ = 91%) (Fig. 5). The differing definitions of stricture response and differing modalities (endoscopic/radiologic) contributed to the heterogeneity. A leave-one-out analysis was performed, and on omitting Aggarwal P 2017, the stricture response was 0.76 [0.65; 0.84] with I^2^ = 0% (Additional file 1: Fig. S7).

### Need for intervention

**Fig. 6:**
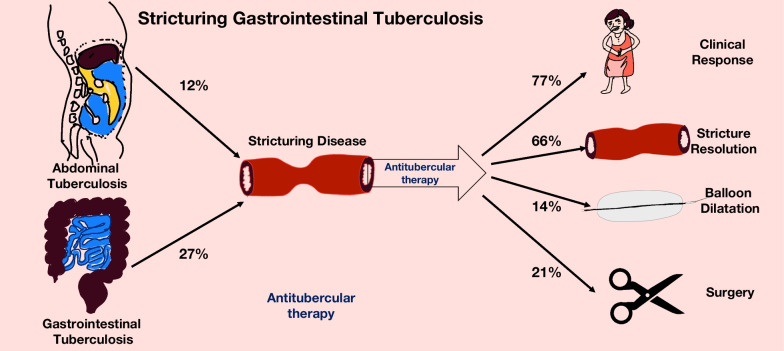
Pictorial depiction of the summary of findings of the systematic review

The pooled rate of surgery (12 studies, 328 patients) was 0.21 (95% CI 0.13–0.32, I^2^ = 70%). The pooled rate of endoscopic dilatation (4 studies, 192 patients) was 0.14 (95%CI 0.09–0.21, I^2^ = 0%) (Fig. 6).

#### Risk of bias

The results of the risk of bias assessment are shown in Additional file 1: Table S4. Of the included studies, six were of good quality, fifteen were of fair quality, and the remaining were of poor quality.

The publication bias for the studies reporting the frequency of stricturing disease in patients of gastro-intestinal TB was assessed using Funnel plot and Eggers’ test** (**Additional file 1: Fig. S8). The Eggers’ test suggests the presence of publication bias (*t* statistic = − 2.91, *p* = 0.007). However, the visual interpretation of the funnel plot suggests a significant horizontal scatter of even the powerful studies suggesting that the results may be due to underlying heterogeneity. The use of trimfill method made the plot more symmetrical but with many studies still outside the funnel. The adjusted pooled estimate of stricturing in abdominal tuberculosis was 0.36 [0.28; 0.44] (with 10 additional ‘missing’ studies).

## Discussion

The results of the present systematic review suggest that stricturing GITB is a significant problem that could be encountered in around a quarter of the patients with gastrointestinal tuberculosis. The findings also suggest that while most patients have a clinical response and improvement in strictures with antitubercular therapy, around 21% of stricturing GITB may need surgical intervention to alleviate the persistent symptoms (Fig. 6). The rates of endoscopic interventions are lower than those of surgery; this may be due to lack of access or feasibility of endoscopic dilatation. Endoscopic dilatation is feasible only in relatively shorter strictures, which can be accessed using colonoscopy.

Gastrointestinal strictures are one of the morphological patterns which are seen in the spectrum of abdominal tuberculosis. They are important, although not the only, cause of abdominal pain and intestinal obstruction in these patients. Other important causes of intestinal obstruction could be mass-forming (pseudo-tumoral or hypertrophic) intestinal tuberculosis, adhesions due to peritoneal involvement, or the formation of abdominal cocoon [1–4, 44, 45]. Nevertheless, strictures are one of the potentially treatable causes of symptoms because part of the pathophysiological processes is potentially reversible. Our analysis demonstrates that symptomatic improvement occurs in most patients with ATT. Some amount of stricture resolution also occurs in most of the patients. However, a complete resolution of the strictures is infrequent. This correlates with the pathophysiological understanding of two dominant phenomena participating in stricture formation: inflammatory narrowing and fibrosing stenosis. Often the tubercular strictures are associated with ulcerations, and with antitubercular therapy, there is a healing of the ulcers [46, 47]. As against the lesions in Crohn’s disease, tubercular ulcers are typically non-penetrating, and the associated edema is also less than in CD. The degree of fibrosis is variable and possibly relates to the duration of the disease process [48]. This suggests that the narrowing may be reversible at least early in the disease course. This would resolve symptoms in most patients, but morphological anomalies may persist.

Although we had planned for analysis of the clinical presentation of stricturing GITB, most of the studies reported the clinical presentation of the entire subset of the GITB (with or without strictures). Nevertheless, most studies suggest abdominal pain and features of intestinal obstruction dominate the clinical presentation of stricturing GITB [11, 13, 31]. The predictors of clinical outcomes, need of endoscopic dilatation or surgery are unclear. In a study by Anand BS et al., young females with longer duration of symptoms were less likely to have a radiological response [11]. The site of the stricture did not seem to impact the outcomes. In contrast, a large recent study from India suggested that colonic strictures are less likely to respond to ATT [13]. Understandably, the resolution rates were also worse in the patients with longer (> 3 cm long) or multiple strictures [13]. The present systematic review also provides estimates of the need for interventions in these patients with around one-fifths of the patients requiring surgery. This suggests that a fraction of the patients might have dominant fibrosis related strictures and do not improve with ATT. It is unclear if preoperative evaluation using imaging could differentiate inflammatory strictures from fibrotic strictures and therefore predict response to ATT. This differentiation has been reported in the setting of CD but not in GITB [49].

Our systematic review has certain limitations: we could not analyze the frequency of the involvement of various sites and response to ATT because of variable definitions of the site and lack of data regarding response (Additional file 1: Table S5). The diagnostic criteria used in various studies were also different and could be responsible for the heterogeneity (Additional file 1: Table S6). We attempted to evaluate high heterogeneity using subgroup analyses and sensitivity analyses, but these could not explain the heterogeneity completely. Also, we did not have data regarding the clinical features, as most studies reported clinical features for the entire subset of GITB. This was because the reporting was variable: some studies reported distal ileum and ileocecal together, while others reported terminal ileum strictures with small bowel. We could not calculate the frequency of clinical symptoms of stricturing GITB as most studies provided clinical features for the entire group of patients with GITB. The impact of the site of involvement on clinical improvement or stricture resolution could also not be estimated because only a few studies provided data separately for resolution rates depending on the site. We had to exclude a large number of studies that provided data only from surgical series because of selection bias towards ‘severely symptomatic’ GITB requiring surgical intervention. In addition, while we have pooled the need for surgery and endoscopic dilatation- the standards for these therapies could be variable between various centers, and the choice of therapy may depend on the local preferences and expertise. The impact of disease duration on the degree of strictures and, eventually, the impact of the degree of strictures on response to ATT may be better estimated by an individual participant meta-analysis with complete details of stricture estimates based on radiological or endoscopic criteria. Because of the heterogeneity in study design, participants, and outcomes estimation, we used a random effects model- however, such a model tends to weigh the study effects more equally and provide more conservative estimates. The study has multiple strengths apart from being the first such analysis of the frequency and impact of stricturing GITB. The analysis included a large number of studies, and we could analyze the frequency of stricturing disease separately for colonic tuberculosis. The study also provided estimates on clinical improvement and stricture response which could help the clinicians in appropriate prognostication of such patients.

Future studies should try to address the issues of heterogenity in disease definitions, study populations, response assessment. This can be accomplished by clear case definitions (microbiologically diagnosed or clinically diagnosed case) of tuberculosis, clear definition of site of involvement, timing of stricture development (symptom duration and relationship with ATT), standard therapy in all cases and clear criteria to define strictures and response (imaging for small bowel and colonoscopy for large intestine) and homogenous assessment of timing of response assessment.

## Conclusion

The present systematic review found that stricturing disease occurs in around a quarter of patients with gastrointestinal tuberculosis. Most patients (three-fourths) have a symptomatic improvement with antitubercular therapy, while the response of strictures is slightly lower (two-thirds). A substantial number of patients require intervention, including endoscopic dilatation or surgical intervention (one-fifth). Although the present systematic review reports these clinically relevant estimates, these should be interpreted cautiously because of the significant heterogeneity in the analyses especially in relation to the pooled prevalence of stricturing disease and clinical response of stricturing disease to ATT.

## Supplementary Information

**Additional file 1.** Supplementary file.

## Data Availability

No new data were created for this manuscript and this meta-analysis used the data available in public domain.

## References

[1] Sharma V, Debi U, Mandavdhare HS, Prasad KK. Tuberculosis and other mycobacterial infections of the abdomen. In: Kuipers EJ. Encyclopedia of gastroenterology (Second Edition). Academic Press 2020; Pp 646–659

[2] Sharma MP, Bhatia V (2004). Abdominal tuberculosis. Indian J Med Res.

[3] Rathi P, Gambhire P (2016). Abdominal tuberculosis. J Assoc Physicians India.

[4] Al-Zanbagi AB, Shariff MK (2021). Gastrointestinal tuberculosis: a systematic review of epidemiology, presentation, diagnosis and treatment. Saudi J Gastroenterol: Off J Saudi Gastroenterol Assoc.

[5] Stroup DF, Berlin JA, Morton SC (2000). Meta-analysis of observational studies in epidemiology: a proposal for reporting. Meta-analysis of observational studies in epidemiology (MOOSE) group. JAMA.

[6] Liberati A, Altman DG, Tetzlaff J (2009). The PRISMA statement for reporting systematic reviews and meta-analyses of studies that evaluate healthcare interventions: explanation and elaboration. BMJ.

[7] Balduzzi S, Rücker G, Schwarzer G (2019). How to perform a meta-analysis with R: a practical tutorial. Evid Based Ment Health.

[8] Viechtbauer W (2022). Conducting meta-analyses in R with the metafor package. J Stat Soft.

[9] Stang A (2010). Critical evaluation of the Newcastle-Ottawa scale for the assessment of the quality of nonrandomized studies in meta-analyses. Eur J Epidemiol.

[10] Egger M, Davey Smith G, Schneider M, Minder C (1997). Bias in meta-analysis detected by a simple, graphical test. BMJ.

[11] Anand BS, Nanda R, Sachdev GK (1988). Response of tuberculous stricture to antituberculous treatment. Gut.

[12] Alvares JF, Devarbhavi H, Makhija P, Rao S, Kottoor R (2005). Clinical, colonoscopic, and histological profile of colonic tuberculosis in a tertiary hospital. Endoscopy.

[13] Aggarwal P, Kedia S, Sharma R (2017). Tubercular intestinal strictures show a poor response to anti-tuberculous therapy. Dig Dis Sci.

[14] Amarapurkar DN, Patel ND, Rane PS (2008). Diagnosis of Crohn's disease in India where tuberculosis is widely prevalent. World J Gastroenterol.

[15] Bhargava DK, Kushwaha AK, Dasarathy S, Chopra P (1992). Endoscopic diagnosis of segmental colonic tuberculosis. Gastrointest Endosc.

[16] Cheng W, Zhang S, Li Y, Wang J, Li J (2019). Intestinal tuberculosis: clinico-pathological profile and the importance of a high degree of suspicion. Trop Med Int Health.

[17] Das HS, Rathi P, Sawant P (2000). Colonic tuberculosis: colonoscopic appearance and clinico-pathologic analysis. J Assoc Physicians India.

[18] **Deka UJ,** Dasgupta JK, Sarkar R, Das K, Banerjee S, Ahmed M, Bhattacharyya A, Basu K. A clinical study of intestinal tuberculosis with special reference to colonic involvement and response to short course anti-tuberculosis therapy. Indian Society of Gastroenterology. Indian J Gastroenterol. 2012; 31:1–114. 10.1007/s12664-012-0264-3

[19] Dutta AK, Sahu MK, Gangadharan SK, Chacko A (2011). Distinguishing Crohn's disease from intestinal tuberculosis–a prospective study. Trop Gastroenterol.

[20] Fillion A, Ortega-Deballon P, Al-Samman S (2016). Abdominal tuberculosis in a low prevalence country. Med Mal Infect.

[21] Gan H, Mely M, Zhao J, Zhu L (2016). An analysis of the clinical, endoscopic, and pathologic features of intestinal tuberculosis. J Clin Gastroenterol.

[22] Hu ML, Lee CH, Kuo CM (2009). Abdominal tuberculosis: analysis of clinical features and outcome of adult patients in southern Taiwan. Chang Gung Med J.

[23] Jung Y, Hwangbo Y, Yoon SM (2016). Predictive factors for differentiating between Crohn's disease and intestinal tuberculosis in Koreans. Am J Gastroenterol.

[24] Kentley J, Ooi JL, Potter J (2017). Intestinal tuberculosis: a diagnostic challenge. Trop Med Int Health.

[25] Khan R, Abid S, Jafri W, Abbas Z, Hameed K, Ahmad Z (2006). Diagnostic dilemma of abdominal tuberculosis in non-HIV patients: an ongoing challenge for physicians. World J Gastroenterol.

[26] Kim KM, Lee A, Choi KY, Lee KY, Kwak JJ (1998). Intestinal tuberculosis: clinicopathologic analysis and diagnosis by endoscopic biopsy. Am J Gastroenterol.

[27] Larsson G, Shenoy KT, Ramasubramanian R (2015). High faecal calprotectin levels in intestinal tuberculosis are associated with granulomas in intestinal biopsies. Infect Dis (Lond)..

[28] Lee YJ, Yang SK, Byeon JS (2006). Analysis of colonoscopic findings in the differential diagnosis between intestinal tuberculosis and Crohn's disease. Endoscopy.

[29] Lu S, Fu J, Guo Y, Huang J (2020). Clinical diagnosis and endoscopic analysis of 10 cases of intestinal tuberculosis. Medicine (Baltimore).

[30] Lu Y, Chen Y, Peng X (2021). Development and validation of a new algorithm model for differential diagnosis between Crohn's disease and intestinal tuberculosis: a combination of laboratory, imaging and endoscopic characteristics. BMC Gastroenterol.

[31] Makanjuola D, al Orainy I, al Rashid R, Murshid K. Radiological evaluation of complications of intestinal tuberculosis. Eur J Radiol. 1998;26(3):261–268. 10.1016/s0720-048x(96)01091-1PMID: 9587753 10.1016/s0720-048x(96)01091-1 9587753

[32] Millar AJW, Rode H, Cywes S (1990). Abdominal tuberculosis in children surgical management. Pediatr SurgInt.

[33] Misra SP, Misra V, Dwivedi M, Gupta SC (1999). Colonic tuberculosis: clinical features, endoscopic appearance and management. J Gastroenterol Hepatol.

[34] Mukewar S, Mukewar S, Ravi R, Prasad A, S Dua K. Colon tuberculosis: endoscopic features and prospective endoscopic follow-up after anti-tuberculosis treatment. Clin Transl Gastroenterol. 2012;3(10):e24. 10.1038/ctg.2012.19PMID: 23238066 10.1038/ctg.2012.19 PMC3491534 23238066

[35] Nagi B, Kochhar R, Bhasin DK, Singh K (2003). Colorectal tuberculosis. Eur Radiol.

[36] Palmer KR, Patil DH, Basran GS, Riordan JF, Silk DB (1985). Abdominal tuberculosis in urban Britain–a common disease. Gut.

[37] Singh H, Krishnamurthy G, Rajendran J (2018). Surgery for abdominal tuberculosis in the present Era: experience from a tertiary-care center. Surg Infect (Larchmt).

[38] Singh V, Kumar P, Kamal J, Prakash V, Vaiphei K, Singh K (1996). Clinicocolonoscopic profile of colonic tuberculosis. Am J Gastroenterol.

[39] Sinha S, Malik S, Kochhar R, Vaiphei K, Sharma K, Koshy A, Berry N, Dhaka N (2017). Narrow band imaging guided biopsy improves yield of histology for diagnosis of gastrointestinal tuberculosis. Gastroenterology.

[40] Tripathi PB, Amarapurkar AD (2009). Morphological spectrum of gastrointestinal tuberculosis. Trop Gastroenterol.

[41] Udgirkar S, Jain S, Pawar S, Chandnani S, Contractor Q, Rathi P (2019). Clinical profile, drug resistance pattern and treatment outcomes of abdominal tuberculosis patients in Western India. Arq Gastroenterol.

[42] Uygur-Bayramicli O, Dabak G, Dabak R (2003). A clinical dilemma: abdominal tuberculosis. World J Gastroenterol.

[43] Zhu QQ, Zhu WR, Wu JT, Chen WX, Wang SA (2014). Comparative study of intestinal tuberculosis and primary small intestinal lymphoma. World J Gastroenterol.

[44] Sharma V, Singh H, Mandavdhare HS (2017). Tubercular abdominal cocoon: systematic review of an uncommon form of tuberculosis. Surg Infect (Larchmt).

[45] Ahamed ZR, Shah J, Agarwala R, Kumar-M P, Mandavdhare HS, Gupta P, Singh H, Sharma A, Dutta U, Sharma V (2019). Controversies in classification of peritoneal tuberculosis and a proposal for clinico-radiological classification. Expert Rev Anti Infect Ther.

[46] Dasgupta A, Singh N, Bhatia A (2009). Abdominal tuberculosis: a histopathological study with special reference to intestinal perforation and mesenteric vasculopathy. J Lab Phys.

[47] Sharma V, Mandavdhare HS, Dutta U (2018). Letter: mucosal response in discriminating intestinal tuberculosis from Crohn's disease-when to look for it?. Aliment Pharmacol Ther.

[48] Tandon HD, Prakash A (1972). Pathology of intestinal tuberculosis and its distinction from Crohn's disease. Gut.

[49] Rimola J, Capozzi N (2020). Differentiation of fibrotic and inflammatory component of Crohn's disease-associated strictures. Intest Res.

