# Supplementary material for: Frequency, outcomes, and need for intervention in stricturing gastrointestinal tuberculosis: a systematic review and meta-analysis
Source: BMC Gastroenterol. 2023 Feb 23;23:46. doi: 10.1186/s12876-023-02682-x (PMC9948355; doi:10.1186/s12876-023-02682-x)

**Supplementary Table 1: Detailed search strategy for the systematic review**

| Pubmed | 13 January 2022 |  |
| --- | --- | --- |
| #1 | Intestinal Tuberculosis OR Gastrointestinal Tuberculosis OR Peritoneal Tuberculosis OR Tuberculous peritonitis OR Abdominal Tuberculosis | 12401 |
| #2 | 'stricture’ OR ‘fibrosis’ OR ‘stenosis’ OR ‘surgery' | 5,546,299 |
| #3= #1 AND #2 | (Intestinal Tuberculosis OR Gastrointestinal Tuberculosis OR Peritoneal Tuberculosis OR Tuberculous peritonitis OR Abdominal Tuberculosis) AND ('stricture’ OR ‘fibrosis’ OR ‘stenosis’ OR ‘surgery') | 4255 |
| Embase | 13 January 2022 |  |
| #1 | 'tuberculous peritonitis' OR 'gastrointestinal tuberculosis' OR 'intestinal tuberculosis' OR 'abdominal tuberculosis' | 5759 |
| #2 | stricture'/exp OR 'stricture' OR 'fibrosis'/exp OR 'fibrosis' OR 'stenosis'/exp OR 'stenosis' OR 'surgery'/exp OR surgery | 7848079 |
|  | ('stricture'/exp OR 'stricture' OR 'fibrosis'/exp OR 'fibrosis' OR 'stenosis'/exp OR 'stenosis' OR 'surgery'/exp OR surgery) AND ('tuberculous peritonitis'/exp OR 'tuberculous peritonitis' OR 'gastrointestinal tuberculosis'/exp OR 'gastrointestinal tuberculosis' OR 'intestinal tuberculosis'/exp OR 'intestinal tuberculosis' OR 'abdominal tuberculosis'/exp OR 'abdominal tuberculosis') | 2597 |

**Supplementary Table 2: The studies excluded from the analysis with the reasons of exclusion**

| S.No | Study | Country | No of stricturing patients | Reason for exclusion |
| --- | --- | --- | --- | --- |
| 1 | Arbo A 2010 | Pakistan | 28 | Surgical series |
| 2 | Barot M 2021 | India | 26 | Surgical series, stricture was managed surgically, response to ATT not mentioned |
| 3 | Bellam BL 2019 | India | 10 | No follow up data on response of stricture to treatment |
| 4 | Burke K 2014 | UK | 1 | Retrospective surgical series of TB with acute abdomen, one case with stricture in duodenum, duration of ATT not mentioned |
| 5 | Chalya PL 2013 | Tanzania | 78 | Data on surgical intervention for abdominal TB, ATT duration and response not mentioned |
| 6 | Chalya PL 2014 | Tanzania | 30 | Surgical series on bowel obstruction |
| 7 | Clarke DL 2007 | South Africa | 3 | Abdominal TB presentation in HIV patients, treatment and response not mentioned |
| 8 | Dalal A 2019 | India | 51 | Stricture managed with both ATT and endoscopic dilation, response to endoscopic dilation mentioned but no clear data  for ATT  Data only for gastroduodenal TB |
| 9 | Dawra S 2019 | India | 7 | Series on ATT induced hepatitis in abdominal TB, stricture response to ATT not mentioned |
| 10 | Gill P 2013 | Australia | 4 | Presentation and surgery role discussed, ATT response not mentioned |
| 11 | Goel D 2009 | India | 78 | Abstract, surgical series |
| 12 | Gorsi U 2018 | India | 1 | Retrospective radiological case series on abdominal cocoon, no data on stricture mentioned specifically |
| 13 | Gupta SK 1998 | India | 5 | Radiological case series |
| 14 | Ha HK 1997 | South Korea | 16 | Retrospective Surgical series, radiological and pathological features studied |
| 15 | Homan PW 1977 | USA | 2 | Old case series, different ATT regimen |
| 16 | Imran M 2010 | Pakistan | 1 | Surgical series |
| 17 | Khan IA 2008 | Pakistan | 5 | Surgical Series |
| 18 | Katariya RN 1977 | India | 9 | Surgical series showing response of stricturoplasty as compared to resection and anastomosis |
| 19 | Madhok P 1982 | India | Not available | Paediatric surgical series |
| 20 | Malik AM 2011 | Pakistan | 19 | Study on role of diagnostic laparoscopy |
| 21 | Mandavdhare HS 2019 | India | 45 | Overlapping data |
| 22 | Nagi B 2014 | India | 19 | Radiological series on duodenal tuberculosis, ATT response not studied |
| 23 | Nakano M 2013 | Japan | 40 | Radiological series assessing role of abdominal USG for stricture diagnosis |
| 24 | Nakano M 2015 | Japan | 1 | Role of abdominal USG for stricture diagnosis, not studied ATT response for tubercular stricture |
| 25 | Nguyen VH 2002 | Vietnam | 1 | Surgical series on tubercular intestinal obstruction, response to ATT not mentioned |
| 26 | Ozbey H 2000 | Turkey | Not mentioned | Retrospective paediatric surgical series |
| 27 | Pillay SP 1981 | South Africa | 24 | Case series of colonic stricture, response to ATT was not studied |
| 28 | Pruthi HS 1996 | India | 13 | Case series of colonic stricture, response to ATT was not mentioned |
| 29 | Puri AS 1996 | India | 7 | Case series (<10 cases) on rectal tuberculosis |
| 30 | Puri AS 2012 | India | 12 | Case series of gastroduodenal tuberculosis, ATT along with endoscopic therapy evaluated together as management for stricture |
| 31 | Rahman O 2014 | Bangladesh | Not mentioned | Patients with stricture number not mentioned and indication of surgery is not clear |
| 32 | Saaiq M 2012 | Pakistan | 161 | Covered surgical management response to ATT not mentioned |
| 33 | Sonika U 2017 | India | 24 | Etiology of small bowel strictures, response to. ATT not studied |
| 34 | Wani MU 2012 | India | 5 | Surgical series on Acute abdomen due to tuberculosis, data on stricture responding to ATT not mentioned |

References:

1. Abro A, Siddiqui FG, Akhtar S, Memon AS. Spectrum of clinical presentation and surgical management of intestinal tuberculosis at tertiary care hospital. J Ayub Med Coll Abbottabad. 2010 Jul-Sep;22(3):96-9. PMID: 22338429.
2. Barot M, Yagnik VD, Patel K, Dawka S. Surgical management of abdominal tuberculosis: A prospective single-center study. *Tzu Chi Med J*. 2020;33(3):282-287. Published 2020 Dec 24. doi:10.4103/tcmj.tcmj_206_20
3. Bellam BL, Mandavdhare HS, Sharma K, et al. Utility of tissue Xpert-Mtb/Rif for the diagnosis of intestinal tuberculosis in patients with ileocolonic ulcers. *Ther Adv Infect Dis*. 2019;6:2049936119863939. Published 2019 Aug 7. doi:10.1177/2049936119863939
4. Burke KA, Patel A, Jayaratnam A, Thiruppathy K, Snooks SJ. Diagnosing abdominal tuberculosis in the acute abdomen. *Int J Surg*. 2014;12(5):494-499. doi:10.1016/j.ijsu.2014.02.006
5. Chalya PL, Mchembe MD, Mshana SE, Rambau PF, Jaka H, Mabula JB. Clinicopathological profile and surgical treatment of abdominal tuberculosis: a single centre experience in northwestern Tanzania. *BMC Infect Dis*. 2013;13:270. Published 2013 Jun 8. doi:10.1186/1471-2334-13-270
6. Chalya PL, Mabula JB, Chandika AB, Giiti G. Dynamic bowel obstruction: aetiology, clinical presentation, management and outcome at Bugando Medical Centre, Mwanza, Tanzania. *Tanzan J Health Res*. 2014;16(1):38-46. doi:10.4314/thrb.v16i1.6
7. Clarke DL, Thomson SR, Bissetty T, Madiba TE, Buccimazza I, Anderson F. A single surgical unit's experience with abdominal tuberculosis in the HIV/AIDS era. *World J Surg*. 2007;31(5):1087-1098. doi:10.1007/s00268-007-0402-8
8. Dalal A, Puri AS, Sachdeva S, Sakuja P. Nonsurgical management of gastroduodenal tuberculosis: Nine-year experience from a tertiary referral center. *Endosc Int Open*. 2019;7(10):E1248-E1252. doi:10.1055/a-0957-2754
9. Dawra S, Mandavdhare HS, Singh H, Prasad KK, Dutta U, Sharma V. Extra-abdominal involvement is associated with antitubercular therapy-related hepatitis in patients treated for abdominal tuberculosis. *Clin Exp Hepatol*. 2019;5(1):60-64. doi:10.5114/ceh.2019.83158
10. Gill P, Coatsworth NR, Gundara JS, Hugh TJ, Samra JS. Tuberculosis: experience in a low endemic area Australian tertiary hospital. *World J Surg*. 2013;37(5):984-990. doi:10.1007/s00268-013-1935-7
11. Aigner, Felix. "1st Biennial Congress of the Eurasian Colorectal Technologies Association (ECTA), 13–15 November 2009, Guangzhou, China." *Techniques in Coloproctology* 14.1 (2010): 55-55
12. Gorsi U, Gupta P, Mandavdhare HS, Singh H, Dutta U, Sharma V. The use of computed tomography in the diagnosis of abdominal cocoon. *Clin Imaging*. 2018;50:171-174. doi:10.1016/j.clinimag.2018.03.014
13. Gupta SK, Jain AK, Gupta JP, Agrawal AK, Berry K. Duodenal tuberculosis. *Clin Radiol*. 1988;39(2):159-161. doi:10.1016/s0009-9260(88)80016-3
14. Ha HK, Ko GY, Yu ES, et al. Intestinal tuberculosis with abdominal complications: radiologic and pathologic features. *Abdom Imaging*. 1999;24(1):32-38. doi:10.1007/s002619900436
15. Homan WP, Grafe WR, Dineen P. A 44-year experience with tuberculous enterocolitis. *World J Surg*. 1977;2(1):245-250. doi:10.1007/BF01665095
16. Imran M, Mahmood Z, Shah TA. Spectrum of presentation of abdominal tuberculosis at Ghurki trust teaching hospital, Lahore. Pakistan Journal of Medical and Health Sciences 2010; 4 (3): 236-38
17. Khan IA, Khattak IU, Asif S, Nasir M, Zia-ur-Rehman. Abdominal tuberculosis an experience at Ayub Teaching Hospital Abbottabad. J Ayub Med Coll Abbottabad. 2008 Oct-Dec;20(4):115-8. PMID: 19999221. Katariya RN, Sood S, Rao PG, Rao PL. Stricture-plasty for tubercular strictures of the gastro-intestinal tract. *Br J Surg*. 1977;64(7):496-498. doi:10.1002/bjs.1800640713
18. Katariya RN, Sood S, Rao PG, Rao PL. Stricture-plasty for tubercular strictures of the gastro-intestinal tract. Br J Surg. 1977 Jul;64(7):496-8. doi: 10.1002/bjs.1800640713. PMID: 922310.
19. Madhok P, Kapur VK. Abdominal tuberculosis in children. *Prog Pediatr Surg*. 1982;15:173-180.
20. Malik AM, Talpur KA, Soomro AG, Qureshi JN. Yield of diagnostic laparoscopy in abdominal tuberculosis: is it worth attempting?. *Surg Laparosc Endosc Percutan Tech*. 2011;21(3):191-193. doi:10.1097/SLE.0b013e318218a974
21. Mandavdhare HS, Singh H, Dutta U, Sharma V. A real-world experience with 6 months of antitubercular therapy in abdominal tuberculosis. *JGH Open*. 2019;3(3):201-205. Published 2019 Jan 16. doi:10.1002/jgh3.12136
22. Nagi B, Lal A, Gupta P, Kochhar R, Sinha SK. Radiological findings in duodenal tuberculosis: a 15-year experience. *Abdom Imaging*. 2015;40(5):1104-1109. doi:10.1007/s00261-014-0302-y
23. Nakano M, Oka S, Tanaka S, et al. Clinical usefulness of classification by transabdominal ultrasonography for detection of small-bowel stricture. *Scand J Gastroenterol*. 2013;48(9):1041-1047. doi:10.3109/00365521.2013.822546
24. Nakano M, Oka S, Tanaka S, et al. Clinical usefulness of transabdominal ultrasonography prior to patency capsule for suspected small-bowel strictures. *Scand J Gastroenterol*. 2016;51(3):281-287. doi:10.3109/00365521.2015.1095942
25. Nguyen VH. Intestinal obstruction due to tuberculosis. *Asian J Surg*. 2002;25(2):145-148. doi:10.1016/S1015-9584(09)60163-9
26. Ozbey H, Tireli GA, Salman T. Abdominal tuberculosis in children. *Eur J Pediatr Surg*. 2003;13(2):116-119. doi:10.1055/s-2003-39588
27. Pillay SP, Moshal MG, Spitaels JM, et al. Etiology of colonic strictures in South African black and Indian patients. *Dis Colon Rectum*. 1981;24(2):107-113. doi:10.1007/BF02604298
28. Pruthi HS, Thakur SK. BENIGN COLONIC STRICTURES - A STUDY OF TWENTY CASES. *Med J Armed Forces India*. 1996;52(4):217-220. doi:10.1016/S0377-1237(17)30869-9
29. Puri AS, Vij JC, Chaudhary A, et al. Diagnosis and outcome of isolated rectal tuberculosis. *Dis Colon Rectum*. 1996;39(10):1126-1129. doi:10.1007/BF02081413
30. Puri AS, Sachdeva S, Mittal VV, et al. Endoscopic diagnosis, management and outcome of gastroduodenal tuberculosis. *Indian J Gastroenterol*. 2012;31(3):125-129. doi:10.1007/s12664-012-0203-3
31. Rahman O, Kabir A, Biswas PK, Islam AS, Siddik A, Rahman H. Clinical Presentation and Treatment Outcome of Abdominal Tuberculosis. J. Medicine [Internet]. 2014 Oct. 19 [cited 2022 Jan. 16];15(2):131-4
32. Saaiq M, Shah SA, Zubair M. Abdominal tuberculosis: epidemiologic profile and management experience of 233 cases. *J Pak Med Assoc*. 2012;62(7):704-707.
33. Sonika U, Saha S, Kedia S, et al. Predictive factors for malignancy in undiagnosed isolated small bowel strictures. *Intest Res*. 2017;15(4):518-523. doi:10.5217/ir.2017.15.4.518
34. Wani MU, Parvez M, Kumar SH, Naikoo GM, Jan M, Wani HA. Study of Surgical Emergencies of Tubercular Abdomen in Developing Countries. *Indian J Surg*. 2015;77(3):182-185. doi:10.1007/s12262-012-0755-6

**Supplementary Table 3: Table showing the definition of clinical response and stricture response in various included studies**

| **Reference** | **Clinical Response** |  | **Clinical Cure** |  | **Radiological**  **Endoscopic Response** |  | **Radiological**  **Endoscopic Cure** |  |
| --- | --- | --- | --- | --- | --- | --- | --- | --- |
| Anand BS 1988 | Significant clinical improvement | 31/34 | Completely Symptom Free | 26/34 |  |  | Complete Stricture resolution on Barium examination | 16/23 |
| Alvares JF 2005 |  |  | Clinical improvement without needing additional therapy (surgery) | 8/10 |  |  |  |  |
| Aggarwal P 2017 |  |  | Resolution of symptoms attributed to stricture (pain abdomen/recurrent subacute intestinal obstruction | 52/104 |  |  | Stricture resolution: Absence of any evidence of stricture on either endoscopy or radiology | 25/106 |
| Bhargava DK 1992 | No need for surgery | 7/10 |  |  |  |  |  |  |
| Deka UJ 2012 | Clinical improvement | 7/7 |  |  |  |  |  |  |
| Misra SP 1999 | Clinical improvement with no requirement of surgery | 10/12 |  |  | Some reduction in stricture/ narrowing | 10/12 | Complete improvement in endoscopic stricture | 4/12 |
| Mukewar S 2012 | Symptomatic improvement with no need of surgery | 36/30 |  |  | Complete endoscopic resolution of strictures | 16/21 | Endoscopic improvement in stricture | 17/21 |
| Singh H 2018 | Clinical improvement with no need of intervention | 26/48 |  |  |  |  |  |  |
| Singh V  1996 | Clinical improvement (Improvement in pain, fever, diarrhea, bleeding, appetite and weight) | 17/17 |  |  |  |  |  |  |
| Sinha S 2017 | Endoscopic or radiological stricture with clinical improvement and no need for surgery | 9/13 |  |  |  |  |  |  |
| Udgirkar S 2019 | Symptomatic improvement with no need for intervention | 23/28 |  |  | Any improvement in stricture eon colonoscopy | 21/28 | Complete stricture resolution on colonoscopy | 3/28 |

**Supplementary Table 4: Risk of bias analysis using modified version New-Castle Ottawa scale analysis**

| **Number** | **Study** | **Selection** | | | | **Outcome** | | | **Total** |
| --- | --- | --- | --- | --- | --- | --- | --- | --- | --- |
|  |  | 1  Representativeness of the TB Stricture cohort | 2  Selection of the TB patients without stricture | 3  Ascertainment of TB stricture diagnosis | 4  Demonstration of Stricture not there at start of study (presentation) | 1  Ascertainment of response of stricture | 2  Follow up long enough for stricture response | 3  Adequacy of follow up of TB stricture patients | **🟑** |
| 1. | Anand BS et al, 1998 | **🟑** | - | **🟑** | **🟑** | **🟑** | **🟑** | **🟑** | 6 |
| 2 | Alvares JF et al, 2005 | **🟑** | **🟑** | **🟑** | - | **🟑** | **🟑** | **🟑** | 6 |
| 3 | Aggarwal P et al, 2017 | **🟑** | **🟑** | **🟑** | **🟑** | **🟑** | **🟑** | **🟑** | 7 |
| 4 | Amrapurkar DN et al, 2008 | **🟑** | **🟑** | **🟑** | - | **🟑** | **🟑** | - | 5 |
| 5 | Bhargava DK et al 1992 | **🟑** | **🟑** | **🟑** | - | **🟑** | **🟑** | **🟑** | 6 |
| 6 | Cheng W et al 2019 | **🟑** | **🟑** | **🟑** | - | **🟑** | - | - | 4 |
| 7 | Das HS et al, 2000 | **🟑** | **🟑** | **🟑** | - | **🟑** | - | - | 4 |
| 8 | Deka UJ et al, 2012 | **🟑** | **🟑** | **🟑** | - | **🟑** | **🟑** | - | 5 |
| 9 | Dutta AK et al, 2011 | **🟑** | **🟑** | **🟑** | **🟑** | **🟑** | - | - | 5 |
| 10 | Fillion A et al, 2015 | **🟑** | **🟑** | **🟑** | - | **🟑** | - | - | 4 |
| 11 | Gan H et al, 2016 | **🟑** | **🟑** | **🟑** | - | **🟑** | **🟑** | - | 5 |
| 12 | Hu ML et al, 2009 | **🟑** | **🟑** | **🟑** | - | **🟑** | **🟑** | **🟑** | 6 |
| 13 | Jung Y et al, 2016 | **🟑** | **🟑** | **🟑** | - | **🟑** | - | - | 4 |
| 14 | Kentley J et al, 2017 | **🟑** | **🟑** | **🟑** | - | **🟑** |  | - | 4 |
| 15 | Khan R et al, 2016 | **🟑** | **🟑** | **🟑** | - | **🟑** | **🟑** | **🟑** | 6 |
| 16 | Kim KM et al, 1998 | **🟑** | **🟑** | **🟑** | - | **🟑** | - | - | 4 |
| 17 | Larsson G et al, 2015 | **🟑** | **🟑** | **🟑** | - | **🟑** | - | - | 4 |
| 18 | Lee YJ et al, 2006 | **🟑** | **🟑** | **🟑** | - | **🟑** | - | - | 4 |
| 19 | Lu S et al, 2020 | **🟑** | **🟑** | **🟑** | - | **🟑** | **🟑** | - | 5 |
| 20 | Lu Y et al, 2021 | **🟑** | **🟑** | **🟑** | - | **🟑** | - | - | 4 |
| 21 | Makanjuola D et al, 1998 | **🟑** | **🟑** | **🟑** | - | **🟑** | - | - | 4 |
| 22 | Millar AJW et al, 1990 | **🟑** | - | **🟑** | - | **🟑** | **🟑** | **🟑** | 5 |
| 23 | Misra SP et al, 1999 | **🟑** | **🟑** | **🟑** | **🟑** | **🟑** | **🟑** | **🟑** | 7 |
| 24 | Mukewar S et al, 2012 | **🟑** | **🟑** | **🟑** | **🟑** | **🟑** | **🟑** | **🟑** | 7 |
| 25 | Nagi B et al, 2013 | **🟑** | **🟑** | **🟑** | - | **🟑** | **🟑** | - | 5 |
| 26 | Palmer KR et al, 1985 | **🟑** | **🟑** | **🟑** | - | **🟑** | **🟑** | **🟑** | 6 |
| 27 | Singh H et al, 2018 | **🟑** | **🟑** | **🟑** | **🟑** | **🟑** | **🟑** | **🟑** | 7 |
| 28 | Singh V et al, 1996 | **🟑** | **🟑** | **🟑** | **🟑** | **🟑** | **🟑** | **🟑** | 7 |
| 29 | Sinha S et al, 2017 | **🟑** | **🟑** | **🟑** | - | **🟑** | **🟑** | - | 5 |
| 30 | Tripathi PB et al, 2009 | **🟑** | **🟑** | **🟑** | - | **🟑** | - | - | 4 |
| 31 | Udgirkar S et al, 2019 | **🟑** | **🟑** | **🟑** | **🟑** | **🟑** | **🟑** | **🟑** | 7 |
| 32 | Uygur-Bayramicli O et al, 2003 | **🟑** | **🟑** | **🟑** | - | **🟑** | **🟑** | - | 5 |
| 33 | Zhu QQ et al, 2014 | **🟑** | **🟑** | **🟑** | - | **🟑** | - | - | 4 |

**Supplementary Table 5: Location of intestinal strictures in patients with gastro-intestinal tuberculosis**

| **Authors** | **Number of patients of structuring tuberculosis** | **Site of stricture** | | | | |
| --- | --- | --- | --- | --- | --- | --- |
|  |  | **Duodenum** | **Small intestine** | **Ileo-cecal area** | **Colon** | **Multiple sites** |
| Anand BS 1998 | 39 | 1 | 15 | 10 | 9 | 4 |
| Aggarwal P 2017 | 106 | 3 | 9 | 52 (Distal ileum and ileocecal) | 37 | 5 |
| Khan R 2006 | 17 | - | 11 | - | - | 6 |
| Makanjuola D 1998 | 6 | 1 | 2 | 2 | - | 1 |
| Udgirkar S  2019 | 29 | - | 6 | 13 | 10 | - |

**Supplementary Table 6: Details of the studies with GITB along with the basis of diagnosis**

| **Author** | **GITB patients (n)** | **stricturing TB patients (n)** | **Basis of diagnosis** |
| --- | --- | --- | --- |
| Anand BS et al, 1998 | - | 39 | Stricture radiologically with evidence of acid-fast bacilli on biopsy and/or presence of pulmonary TB |
| Alvares JF et al, 2005 | 43 | 10 | colonoscopy with histological evidence, or evidence of tuberculosis else− where, coupled with a good response to ATT |
| Aggarwal P et al, 2017 | 286 | 128 | -appropriate clinical setting with the demonstration of necrotizing granulomas on histopathology or demonstration of AFB on histopathology or culture -patient had clinical and endoscopic/radiologic response to anti-tubercular treatment. |
| Amrapurkar DN et al, 2008 | 26 | 5 | (1) presence of caseating granuloma on histology of diseased tissue (intestine, peritoneum or lymph nodes) (2) demonstration of AFB on smear or on histological section (3) positive culture for AFB (4) histological or microbiological confirmed TB at extra-intestinal site; (5) positive TB PCR |
| Bhargava DK et al 1992 | 29 | 10 | -either confirmed by histologic evidence of caseating or non-caseating granuloma or a positive culture for mycobacteria -Other criteria included associated focus of tuberculosis elsewhere or an absence of other diseases on biopsies from the endoscopic lesions with a response to ATT. |
| Cheng W et al 2019 | 49 | 11 | (i) caseating or necrotising granulomas upon histological examination (ii) AFB positive in smears or histological sections (iii) AFB-positive culture in a specimen obtained from the lesion site (iv) AFB-positive TB-PCR results obtained from the lesion site (v) complete resolution of symptoms after standard ATTfor 6 weeks |
| Das HS et al, 2000 | 21 | 3 | bacteriological and histological assessment of tissue |
| Deka UJ et al, 2012 | 44 | 7 | clinical, radiological, endoscopic and histopathological findings |
| Dutta AK et al, 2011 | 24 | 4 | (I)Intestinal mucosal biopsy showing –  a) AFB positive on histopathology or culture, and/ or  b) caseating granulomas, and/or  c) large or confluent granulomas  (II) Response to treatment |
| Fillion A et al, 2015 | 7 | 1 | -clinical data (abdominal pain, altered bowel habit, and clinical ascites) and/or imaging features of abdominal infection. -confirmed by positive culture or PCR of *Mycobacterium tuberculosis* complex from histopathology showing a tubercular granuloma (with or without necrosis) in the absence of an alternative diagnosis. |
| Gan H et al, 2016 | 81 | 16 | (1) Presence of caseous necrotizing granulomas on the intestinal wall or in the mesenteric lymph nodes (2) presence of M. tuberculosis in histopathologic examination of lesion tissue (3) Culture-positive M. tuberculosis in a specimen obtained from the lesion site (4) TB changes in animals inoculated with a specimen obtained from a lesion site; and  (5) the presence of typical TB changes by x-ray examination, extraintestinal TB lesions, clinical symptoms and signs, and improvement after anti-TB treatment for 6 weeks. |
| Hu ML et al, 2009 | 3 | 1 | (1) positive culture of *M. tuberculosis*  (2) positive AFB stain from the tissue biopsies (3) histopathological demonstration of caseating granulomatous necrosis (4) positive PCR for *M. tuberculosis* on abdominal organ tissue or peritoneal fluid |
| Jung Y et al, 2016 | 98 | 9 | (i) presence of caseating granuloma on histological examination of diseased tissue  (ii) demonstration of AFB on smears or histological sections (iii) positive culture for AFB (iv) histologically or microbiologically confirmed TB at an extra-intestinal site, and  (v) positive TB-PCR. |
| Kentley J et al, 2017 | 61 | 9 | clinical, radiological, bacteriological and histological findings |
| Khan R et al, 2006 | 102 | 17 | (1) positive AFB smear or culture (2) histopathology showing tubercular granuloma (with or without caseation),  (3) radiological features compatible with tuberculosis (4) patients with a high index of clinical suspicion and negative diagnostic workup but showed a good response to therapeutic trial of ATT |
| Kim KM et al, 1998 | 42 | 3 | Colonoscopy guided biopsy +PCR positivity |
| Larsson G et al, 2015 | 30 | 3 | -endoscopic and histologic criteria for ITB were applied to establish the diagnosis -Clinical improvement after a 2-week trial of ATT |
| Lee YJ et al, 2006 | 44 | 8 | a) histologic evidence of caseating granulomas b) histologic demonstration of acid-fast bacilli c) growth of M. tuberculosis on tissue culture d) clinical, endoscopic, radiologic, and/or operative evidence of intestinal tuberculosis with proved tuberculosis elsewhere e) response to ATT without subsequent recurrence in patients |
| Lu S et al, 2020 | 10 | 6 | clinical, radiologic, endoscopic and histological features |
| Lu Y et al, 2021 | 84 | 24 | (a) caseating granulomas detected in endoscopy biopsy, surgical specimen, or mesenteric lymph nodes (b) demonstration of AFB on smears or histological sections or positive culture for AFB (c) strong suspicion of ITB with a good response to ATT without recurrence; a good response to ATT was determined by relief of symptoms and disappearance of ulcerations on endoscopic examination for at least 6 months of follow-up |
| Makanjuola D et al, 1998 | 21 | 8 | Clinical, Endoscopic, Histology and Radiological |
| Misra SP et al, 1999 | 50 | 12 | presence of AFB on histology;  presence of caseating or non-caseating well-formed granuloma(s); associated active pulmonary tuberculosis;  or by the absence of other disease on histological examination along with a good response to ATT |
| Mukewar S et al, 2012 | 69 | 30 | ‘‘confirmed colon TB’’ was made if the biopsy specimen showed caseating granulomas and/or AFB. |
| Nagi B et al, 2003 | 74 | 40 | positive histology/bacteriological examination of gastrointestinal tract lesion, chest manifestations with sputum positive for AFB, histology or a combination of clinical/radiological features with positive response to ATT |
| Palmer KR et al, 1985 | 42 | 10 | Histological examination of tissue obtained by laparotomy & colonoscopy,  Associated with a prompt response to ATT |
| Singh H et al, 2018 | 75 | 48 | 1) AFB on smear or culture of biopsies  (2) caseating granuloma on histologic evaluation in operative or pre-operative tissue or  (3) classical intra-operative findings of tuberculosis with suggestive histology (granulomas) in the resected area |
| Singh V et al, 1996 | 62 | 17 | Colonoscopy appearance, histopathology, active pulmonary TB and response to ATT |
| Sinha S et al, 2017 | 32 | 13 | AFB in tissue or grown in culture, histopathology showed caseous necrosis with granulomatous inflammation or clinical/radiological and endoscopic features were suggestive of tuberculosis and a good clinical response to ATT |
| Tripathi PB et al, 2009 | 110 | 57 | 1. presence of AFB on histology, 2. caseating or non-caseating epithelioid cell granulomas on histology, 3. evidence of TB at other extraintestinal sites seen on radiological imaging  4. all of these along with a complete response to ATT |
| Udgirkar S et al, 2019 | 162 | 29 | clinical profile and supported by gross morphological findings at endoscopy, and/or diagnostic laparoscopy (when needed), followed by histology and/or GeneXpert and MGIT culture |
| Uygur-Bayramicli O et al, 2003 | 19 | 1 | Clinical, Radiological, Endoscopic evaluation, Microbiological, Histology and Response to ATT |
| Zhu QQ et al, 2014 | 35 | 22 | Established clinical, CT, histological and microbiological criteria |

AFB: Acid fast bacilli, ATT: Antitubercular therapy, TB: Tuberculosis, PCR: Polymerase Chain Reaction

**Supplementary Figure 1: Forest Plot showing the pooled prevalence of stricturing disease in patients with abdominal tuberculosis**

**
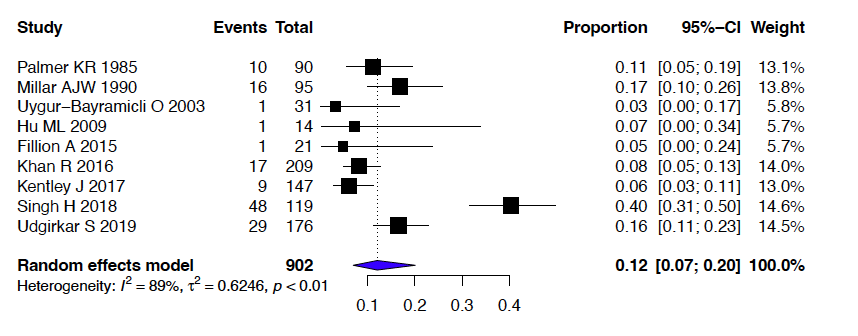
**

**Supplementary Figure 2: Baujat plot showing the studies contributing to heterogeneity**


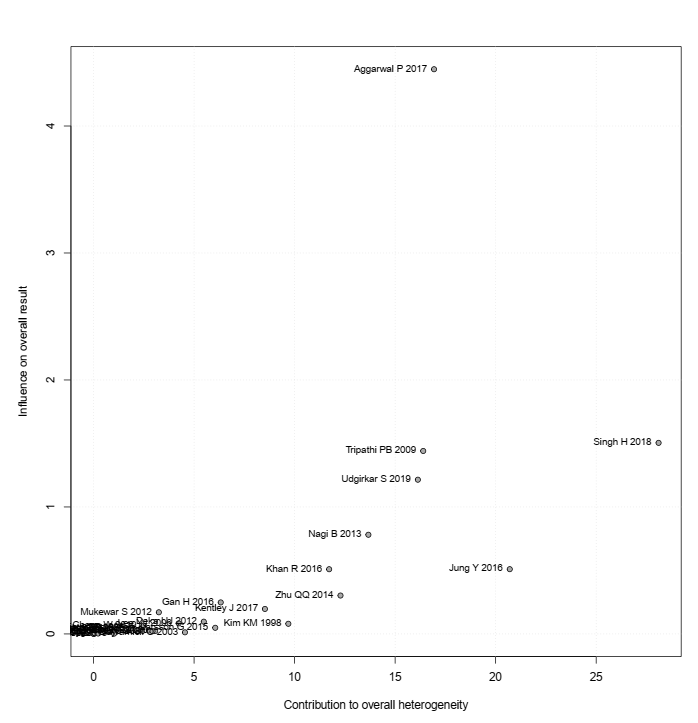


**Supplementary Figure 3: Forest plot showing the frequency of stricturing disease in gastrointestinal tuberculosis stratified by location i.e intestinal or colonic**


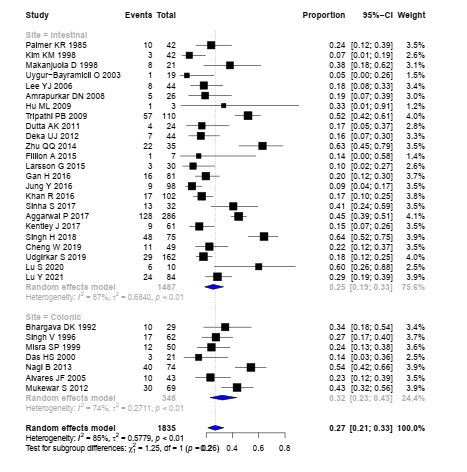


**Supplementary Figure 4: Forest plot showing the subgroup analyses based on the type of study**

**
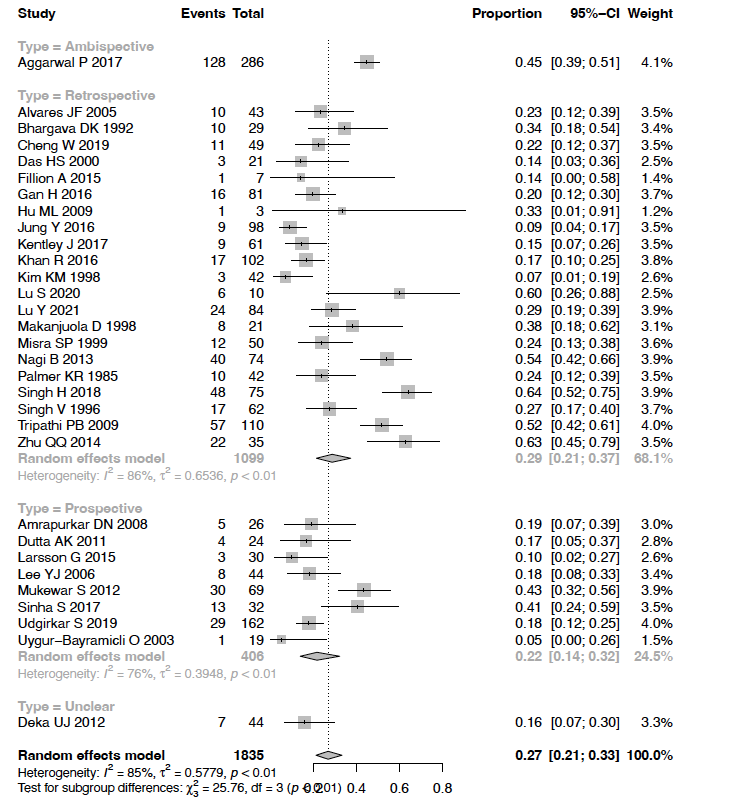
**

**Supplementary Figure 5: Forest plot showing the subgroup analyses based on the duration of the antitubercular therapy**

**
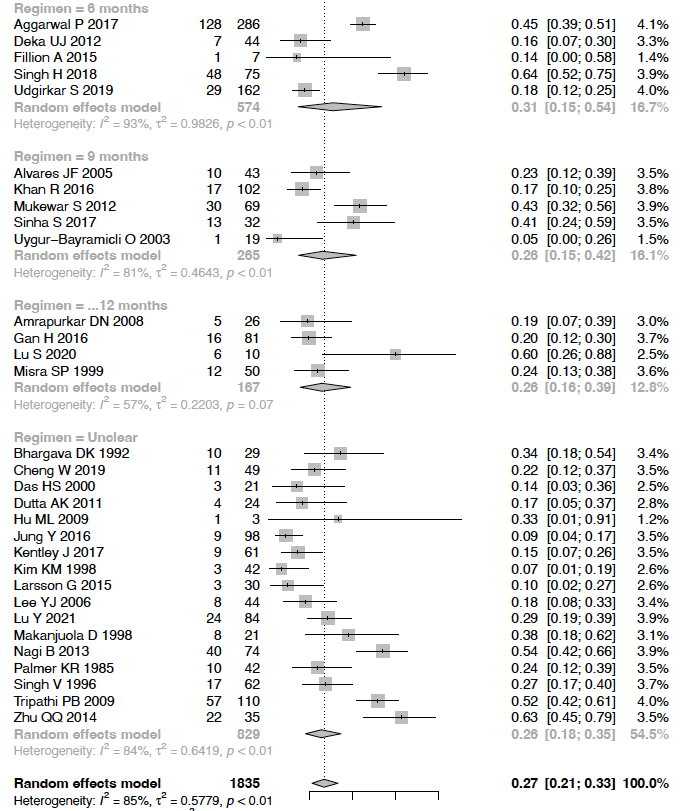
**

**Supplementary Figure 6: Forest plot showing the frequency of stricturing disease in gastrointestinal tuberculosis in studies at a low risk of bias**


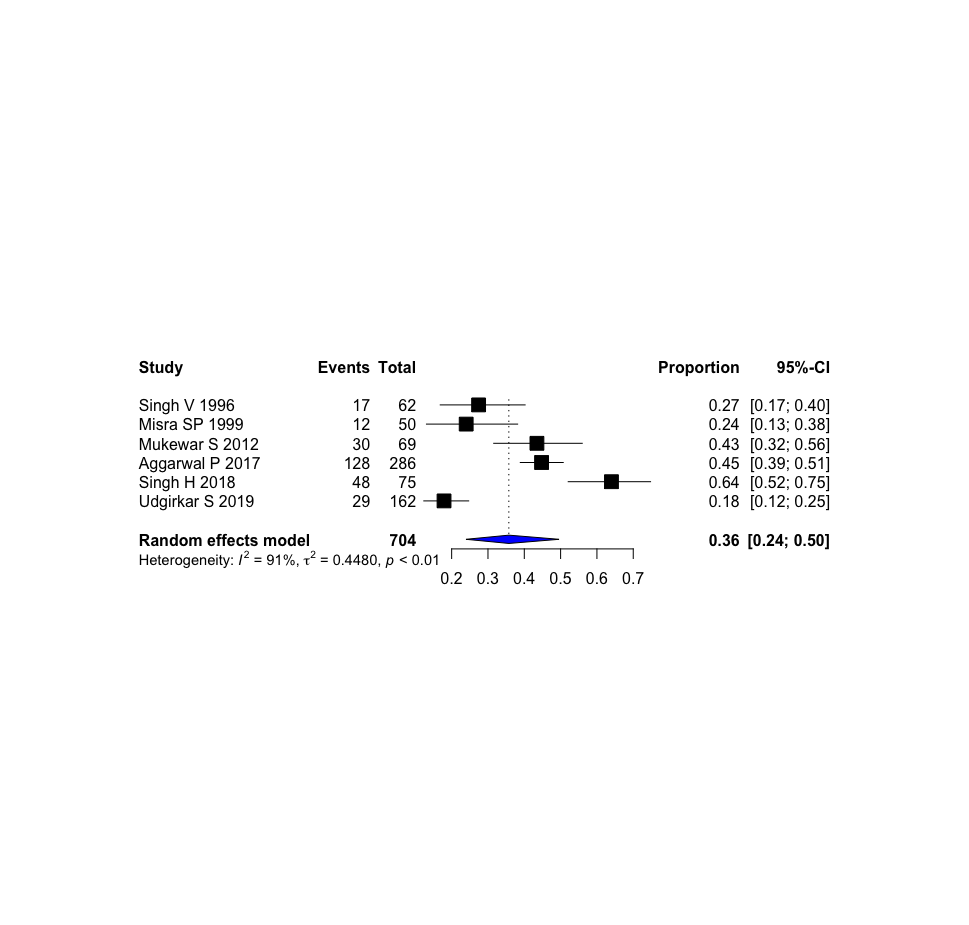


Test for subgroup differences (random effects model):

Q d.f. p-value

Between groups 0.26 3 0.9677

**Supplementary Figure 7: Leave one out analysis of studies reporting stricture response to ATT**

**
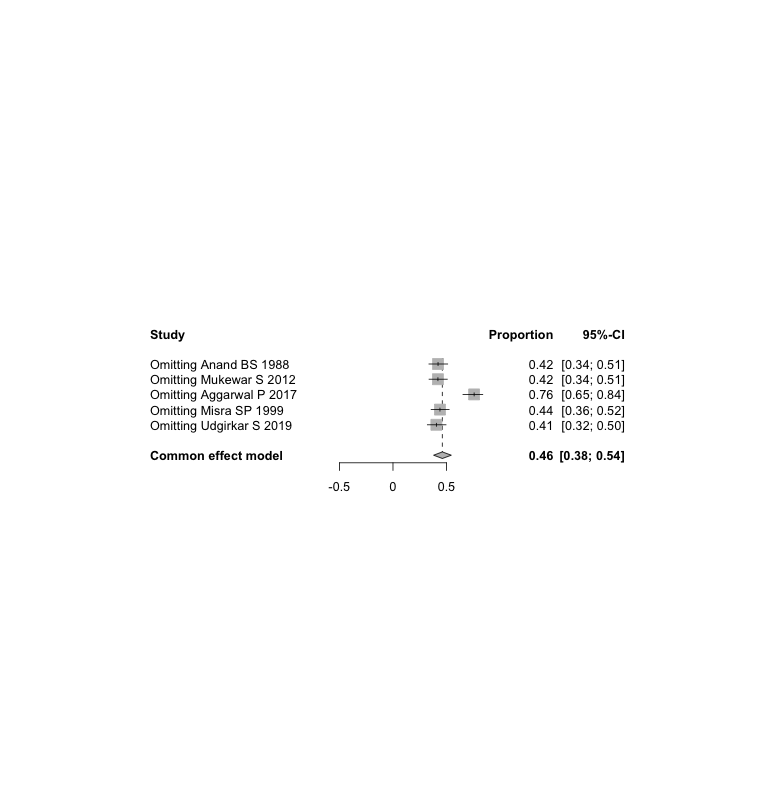
**

Influential analysis (Fixed effect model)

Proportion 95%-CI tau^2 tau I^2

Omitting Anand BS 1988 0.4210 [0.3360; 0.5109] 1.6480 1.2837 92.7%

Omitting Mukewar S 2012 0.4199 [0.3372; 0.5074] 1.3976 1.1822 91.9%

Omitting Aggarwal P 2017 0.7581 [0.6540; 0.8386] 0.0000 0.0000 0.0%

Omitting Misra SP 1999 0.4389 [0.3567; 0.5245] 1.3449 1.1597 92.7%

Omitting Udgirkar S 2019 0.4054 [0.3213; 0.4954] 1.5737 1.2545 91.8%

Pooled estimate 0.4613 [0.3797; 0.5450] 1.2294 1.1088 91.4%

**Supplementary Figure 8: A) Standard Funnel Plot depicting the visual assessment of publication bias B) Funnel plot by Trimfill method**


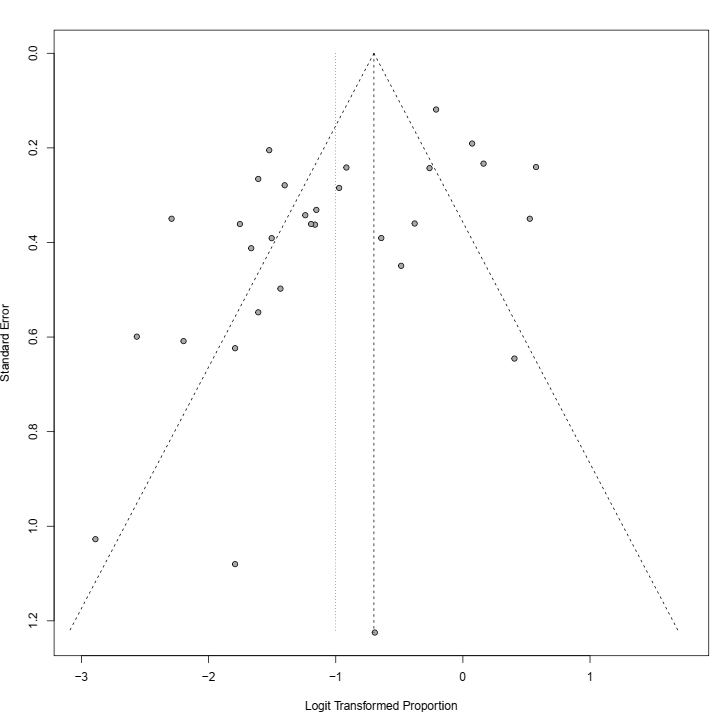

Supplement: Supplementary file 1 — Additional file 1. Supplementary file. [file 12876_2023_2682_MOESM1_ESM.docx]
